# Intrinsically Magnetic Cells: A Review on Their Natural Occurrence and Synthetic Generation

**DOI:** 10.3389/fbioe.2020.573183

**Published:** 2020-10-19

**Authors:** Alexander Pekarsky, Oliver Spadiut

**Affiliations:** Institute of Chemical, Environmental and Bioscience Engineering, Research Area Biochemical Engineering, Technische Universität Wien, Vienna, Austria

**Keywords:** intrinsic magnetism, magnetic cells, magnetotactic bacteria, magnetic protein, ferritin, encpasulin, continuous cultivation

## Abstract

The magnetization of non-magnetic cells has great potential to aid various processes in medicine, but also in bioprocess engineering. Current approaches to magnetize cells with magnetic nanoparticles (MNPs) require cellular uptake or adsorption through *in vitro* manipulation of cells. A relatively new field of research is “magnetogenetics” which focuses on *in vivo* production and accumulation of magnetic material. Natural intrinsically magnetic cells (IMCs) produce intracellular, MNPs, and are called magnetotactic bacteria (MTB). In recent years, researchers have unraveled function and structure of numerous proteins from MTB. Furthermore, protein engineering studies on such MTB proteins and other potentially magnetic proteins, like ferritins, highlight that *in vivo* magnetization of non-magnetic hosts is a thriving field of research. This review summarizes current knowledge on recombinant IMC generation and highlights future steps that can be taken to succeed in transforming non-magnetic cells to IMCs.

## Introduction

**FIGURE 1:**
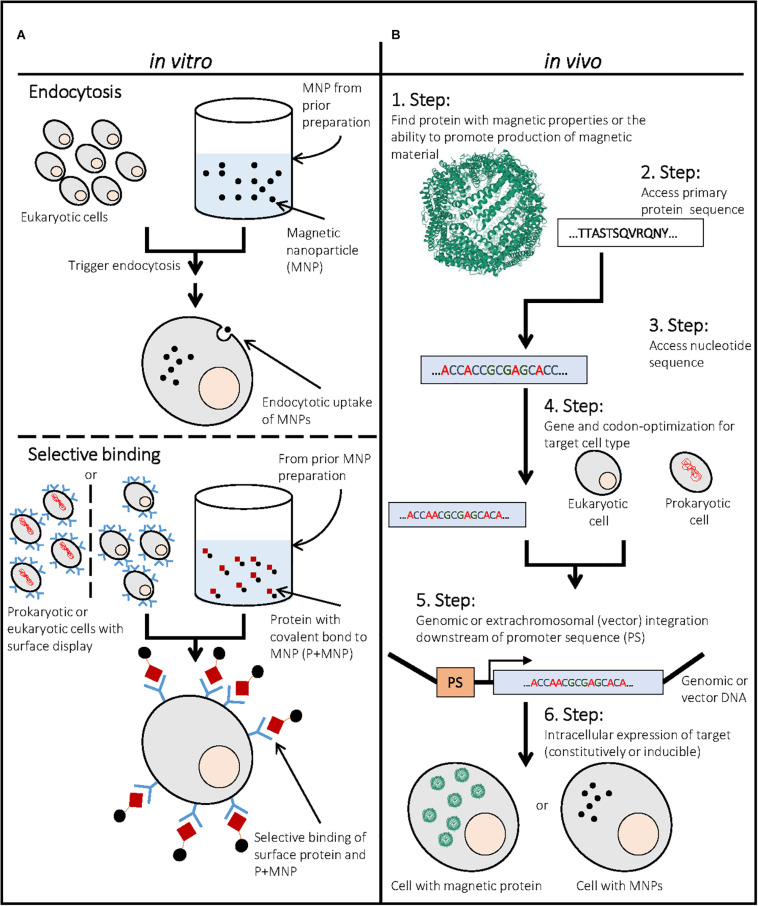
Generation of magnetic cells by *in vitro* and *in vivo* approaches. **(A)** Upper part: Example for the generation of magnetic cells by endocytosis of magnetic nanoparticles (MNPs) with eukaryotic cells. MNPs have to be prepared in a prior step to promote endocytosis. **(A)** Lower part: Example for the generation of magnetic cells through the selective binding of a protein with covalently bound MNP to another cell surface protein (e.g., surface display of antibody that binds MNP-bound antigen). This approach can be performed for eukaryotic and prokaryotic cells by expressing and targeting a respective protein to the cell surface. The MNPs have to be chemically modified to covalently bind a respective antigen, preferably through a covalent bond. **(B)** Example for the generation of intrinsically magnetic cells through expression of a respective protein in the cell. In this case, the iron oxide containing protein ferritin [PDB ID: 1FHA (Lawson et al., 1991)] is seen as an example. First, a respective protein that confers a magnetic moment or is able to generate magnetic precipitates in the cell has to be identified. Further its primary sequence and nucleotide sequence have to be assessed and optimized. Finally, the respective protein can be integrated in a vector or genomic DNA of the cell and expressed to yield intrinsically magnetic cells that contain high amounts of the respective magnetic protein or a magnetic precipitate.

Magnetic nanoparticles (MNPs) have been of importance since the successful use of magnetically labeled antibodies for cell separation (Whitesides et al., 1983; Kemsheadl and Ugelstad, 1985). Since then, magnet-assisted cell separation (MACS) has been frequently used for detection and isolation of cells from complex mixtures [e.g., (McCloskey et al., 2003; Kuhara et al., 2004; Zborowski and Chalmers, 2011)]. The use of MNPs is also of great interest as magnetic resonance imaging (MRI; Li et al., 2016) and magnetic particle imaging (MPI) contrast agent (Kraupner et al., 2017), for targeted drug delivery (Huang et al., 2016; Price et al., 2018), stem cell-based regenerative medicine (Van de Walle et al., 2020), targeted cell delivery (Wu L. et al., 2018), hyperthermia treatment (Kobayashi, 2011; Liu et al., 2020), and magneto-mechanical cell fate regulation (Wu C. et al., 2018). In these approaches, cells take up or adhere MNPs (Figure 1A). Recent research showed that internalized MNPs can undergo degradation (Van de Walle et al., 2019; Curcio et al., 2020). Interestingly, cells showed re-magnetization after MNP degradation, which demonstrated that some cells might contain quiescent abilities to magnetize *in vivo*. Furthermore, there exist prokaryotic organisms that naturally magnetize themselves through intracellular MNP formation (Blakemore, 1975). This existence of natural, cellular magnetization initiated research to uncover important prerequisites for cell magnetization. “Magnetogenetics” focuses on intracellular production and accumulation of magnetic material in non-magnetic cells *in vivo* (Meister, 2016; Nimpf and Keays, 2017; Figure 1B). Several reviews describe the accumulation (Li et al., 2011; Singh et al., 2016; Gorobets et al., 2017; Gahlawat and Choudhury, 2019) and current applications of MNPs (Plouffe et al., 2015; Li et al., 2016; Mohammed et al., 2017; Vargas et al., 2018; Wu et al., 2019; Van de Walle et al., 2020). However, no overview on the generation and application of whole magnetic cells is available. This review specifically discusses intrinsically magnetic cells (IMCs) and recombinant approaches to generate them.

## Magnetism

**FIGURE 2:**
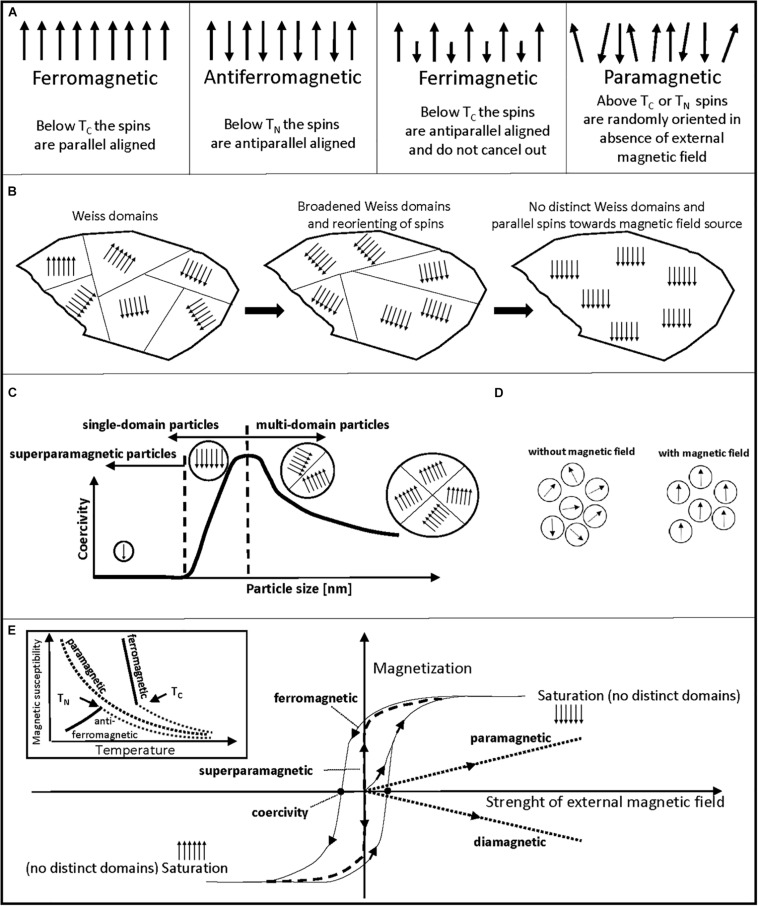
Schematic description of different magnetic properties. **(A)** Schematic orientation of magnetic moments (spins) of ferromagnetic, antiferromagnetic, ferrimagnetic, and paramagnetic materials are shown. **(B)** Broadening and dissipation of Weiss domains of a ferromagnetic material through an increasing, external magnetic field is shown from left to right. **(C)** Schematic graph of coercivity versus particle size. Superparamagnetic particles show no coercivity, coercivity increases for single-domain particles with increasing size until a maximum, when a switch to the energetically more favorable multi-domain state occurs. **(D)** Schematic sample of superparamagnetic particles is shown. Without an external magnetic field, particles show random orientation that leads to no net magnetization of the sample. With an external magnetic field, magnetic moments orient parallel toward the source of the magnetic field. **(E)** Schematic depiction of magnetization curves and their extractable information on magnetic properties of a sample. Temperature (inlet) and magnetic field strength dependency in SQUID magnetometry measurements is shown. Ferro- and ferrimagnetic materials usually show similar curves, only ferromagnetism is shown. Inlet: Magnetic susceptibility versus temperature graph is shown. Based on the changing magnetic susceptibility at rising temperature, one can differentiate antiferromagnetic, ferromagnetic, or paramagnetic properties of a sample. Magnetic susceptibility decreases exponentially with increasing temperature for paramagnetic samples. Until T_*N*_, antiferromagnetic samples show an increase in magnetic susceptibility with temperature. Near T_*C*_, ferro- and ferrimagnetic samples show a steep decrease in magnetic susceptibility with temperature. Then, above T_*N*_ and T_*C*_ paramagnetic behavior is visible for ferro-, ferri-, and antiferromagnetic samples. Main graph: Sample magnetization versus strength of external magnetic field. Non-magnetized samples start at zero magnetization without an external magnetic field. Measurement allows to find coercivity points as marked for hysteresis curve of ferro- and ferrimagnetic samples that arises from moving/dissipating Weiss domains with magnetic field strength. No coercivity and hysteresis is usually visible for superparamagnetic particles. A linear course of sample magnetization is common for paramagnetic and diamagnetic samples. Plateau of sample magnetization marks saturation magnetization of sample, where no distinct Weiss domains exist in multi-domain state particles and is used as a mean to compare magnetic potency of a sample.

Intrinsically magnetic cells are magnetic cells, therefore a brief overview on magnetism and important characteristics in the nano- and micrometer range is given. Due to availability, biocompatibility and magnetic properties, we focus on the element iron. There are different types of magnetism: depending on the magnetic moments that arise from electrons and their two spin states, a material can show ferromagnetism, antiferromagnetism and ferrimagnetism through unpaired electrons (unpaired spins; Figure 2A) or diamagnetism through paired electrons (paired opposite spins). Diamagnetic molecules, like water, are commonly classified as non-magnetic materials and are repelled by strong magnetic fields. In paramagnetic materials, randomly oriented spins are found (Figure 2A; O’Handley, 2000).

Above a certain temperature, called Curie-temperature (T_*C*_) and Néel-temperature (T_*N*_), the magnetic behavior of materials can change to paramagnetism. Metallic iron is ferromagnetic at room temperature and shows equal and aligned magnetic moments below T_*C*_. In antiferromagnetic materials, like hematite (α-Fe_2_O_3_), the spins show opposing directions, but equal magnetic moments. In ferrimagnetic materials, like magnetite (Fe_3_O_4_), maghemite (γ-Fe_2_O_3_) or greigite (Fe_3_S_4_), unequal magnetic moments and opposing directions are present (Figure 2A; O’Handley, 2000). Greigite has a similar structure to magnetite, but with sulfur instead of oxygen ions (Roberts et al., 2011). Reported saturation magnetization values at room temperature are 218 Am^2^ kg^–1^ for ferromagnetic iron (Wohlfarth, 1986), 92–100 Am^2^ kg^–1^ for magnetite, 60–80 Am^2^ kg^–1^ for maghemite, 59 Am^2^ kg^–1^ for greigite and 0.3 Am^2^ kg^–1^ for hematite (Cornell and Schwertmann, 2003; Chang et al., 2008).

The magnetic behavior is not only affected by temperature, but also by particle size. Above a critical particle size, an energetically favorable division of ferro-, ferri-, and antiferromagnetic materials into microscopic domains occurs, called Weiss domains (Figure 2B). These domains show different spin orientations, which result in a low net magnetic field of a macroscopic sample (O’Handley, 2000). When an external magnetic field is applied, these Weiss domains broaden and the spins align, which leads to magnetic attraction and dissipation of the domains (Figure 2B). Additionally, below a certain particle size, the Weiss domains also dissipate to form one single domain (single-domain state). This size-dependency is also visible through coercivity (demagnetization point) measurements in sensitive magnetometry experiments. In the single-domain state not only Weiss domains dissipate, but the external magnetic field required for demagnetization also decreases (Figure 2C). The characteristic size for a certain magnetic material to form a single-domain state differs. For example, a switch to single-domain state was reported below 73 nm for synthetic magnetite crystals (Li et al., 2017). Additionally, some materials exhibit superparamagnetism below 20 nm and above a certain temperature (blocking temperature). Exemplary sizes for superparamagnetic particles are 10 nm for cube-like magnetite (Li et al., 2017), 15–18 nm for maghemite (Ali K. et al., 2015), 16 nm for hematite (Bødker et al., 2000), and 9–14 nm for greigite (Lyubutin et al., 2013). Superparamagnetic particles show a fixed magnetic moment that varies only in direction, have no coercivity and a sample of such particles will have an overall zero net magnetization without an external magnetic field, due to random orientation of particles (Figure 2D). Since superparamagnetic particles also show high initial magnetic susceptibility, only low fields are needed for magnetization, making these particles valuable in medical and biotechnological approaches (Huber, 2005).

Several techniques are currently employed to investigate magnetic materials (Nisticò et al., 2020): superconducting quantum interference device (SQUID) magnetometry (Jenks et al., 1997; Sawicki et al., 2011) and vibrating sample mode (VSM) magnetometry (Foner, 1959) to gain magnetization curves, X-ray diffraction (XRD) to identify the atomic and molecular structure of crystals (Warren, 1990; Mos et al., 2018), and Mössbauer spectroscopy to gain valence state of iron, type of coordination polyhedron occupied by iron atoms and identification of iron oxide phases (Gütlich and Schröder, 2012; Keune, 2012; Kamnev and Tugarova, 2017). SQUID magnetometry is often the method of choice to determine if a sample is ferro-, ferri-, antiferro-, para-, or diamagnetic through measurement at different temperatures and external magnetic field strengths (Jenks et al., 1997; Sawicki et al., 2011). A schematic depiction of SQUID graphs and their extractable magnetic information is given in Figure 2E. For example, variation in temperature allows to distinguish between ferro-/ ferri-, antiferro-, and paramagnetic samples (inlet; Figure 2E). A variation in external magnetic field strength allows one to distinguish between ferro-/ ferri-, para-, dia-, and superparamagnetic samples (Figure 2E). This magnetization to field strength analysis can be also used to determine coercivity points and the saturation magnetization of ferro- and ferrimagnetic samples (Sorensen, 2001).

## General Considerations for *in vivo* Magnetization of Cells

We define an IMC as a cell that (i) exhibits a measurable magnetic moment, (ii) changes its flow direction in a magnetic field, and (iii) has not acquired these properties through uptake of – or surface modification with – MNPs. Living cells mainly consist of water, thus they are usually classified as diamagnetic. However, diamagnetism can be overcome (O’Handley, 2000). One might ask (1) the type and (2) the amount of magnetic material to overcome diamagnetism.

In respect of type, iron is the element of choice, due to its availability, biocompatibility, and magnetic properties. Furthermore, prokaryotic and eukaryotic cells have integrated iron homeostasis (Crichton et al., 2002; Konhauser et al., 2011). In a cell, iron is never present in its metallic form, but in its cationic forms [ferrous (Fe^2+^), ferric (Fe^3+^)] with critical functions in cellular processes. Under physiological conditions only 10^–18^ M soluble Fe^3+^, but 10^–1^ M soluble Fe^2+^ can be accumulated (Bou-Abdallah, 2010). Unpaired electrons are needed for magnetic attraction and a brief look on the electron configuration of Fe^2+^ ([Ar]3d^6^) with 4 unpaired electrons and Fe^3+^ ([Ar]3d^5^) with 5 unpaired electrons might indicate that generation of IMCs is already feasible by intracellular accumulation of soluble iron cations. However, most intracellular iron is complexed by proteins, since accumulation of soluble iron is cytotoxic (Imlay, 2008; Yamada et al., 2012). Since iron cofactor proteins present limited options for cell magnetization, MNPs are a better strategy to magnetize cells – through uptake [e.g., (Van de Walle et al., 2020)] or adsorption [e.g., (McCloskey et al., 2003)]. However, IMCs need to produce MNPs *in vivo* through precipitation and crystallization of magnetic iron oxides (e.g., magnetite) or sulfides (e.g., greigite). Due to its strong magnetization, magnetite has been the material of choice for that purpose.

In respect to the required amount of magnetic material to overcome diamagnetism, it is difficult to state generally valid minimum quantities. The magnetic force and three additional forces are directly proportional to the radius of the respective cell (McCloskey et al., 2003; Plouffe et al., 2015; Zahn et al., 2017). Opposite to the direction of the magnetic force, a viscous drag force slows the cell down. Additionally, the gravitational force and the buoyancy force act on the cell. Calculations for MNP uptake (Plouffe et al., 2015) and adsorption (McCloskey et al., 2003) showed that increasing cell size requires more magnetic material to gain comparable magnetic properties. Furthermore, concerning the size of the intracellular magnetic material the characteristics of superparamagnetic and single-domain state magnetic behavior have to be considered. Recent calculations showed that MNP arrangements influence the magnetic state (Muxworthy and Williams, 2009). The authors showed that chain-like arrangement increases the range of the single-domain state to 12–197 nm for touching cubic-like magnetite particles.

Many molecules and ions that are of utmost importance for the cellular machinery, have small molecular magnetic moments, due to their nuclear spins. Despite the small value of these magnetic moments, a high gradient magnetic field might act on them (Zablotskii et al., 2016a). Thus, magnetic forces might compete and interfere with electrical forces (Zablotskii et al., 2016a). Several studies were performed on a variety of cells to analyze the impact of magnetism on cells. For example, it was long hoped to use high magnetic fields to reduce microbial growth for sanitization. However, neither static nor pulsed magnetic fields showed a significant impact on microbial growth (Harte et al., 2001), viability (László and Kutasi, 2010), or endospore germination (Wu et al., 2017). It is still noteworthy that transposition and heat shock protein activity was induced in *Escherichia coli* cells when exposed to magnetic fields (Chow and Tung, 2000; Potenza et al., 2004; Del Re et al., 2006). Low-level static magnetic fields significantly increased superoxide dismutase and peroxidase activity in suspension-cultured tobacco plant cells (Abdolmaleki et al., 2007; Sahebjamei et al., 2007). A variety of effects were found for mammalian cells. An *in vitro* study on human lymphocytes and macrophages showed morphological changes in an inhomogeneous static magnetic field (Vergallo et al., 2014). In an *in vivo* study with human endothelial cells, proliferation was inhibited and angiogenesis impaired by magnetic field gradients around 2 T m^–1^ (Wang et al., 2009). However, a static homogenous magnetic field (370 mT) showed no impact on human lung fibroblast cells (Romeo et al., 2016). Recent reviews (Zablotskii et al., 2016a,b) highlight that cellular parameters are rather dependent on the value of the magnetic field gradient, but not on the strength of the magnetic field.

## Currently Known Natural IMCs

### Magnetotactic Bacteria

**FIGURE 3:**
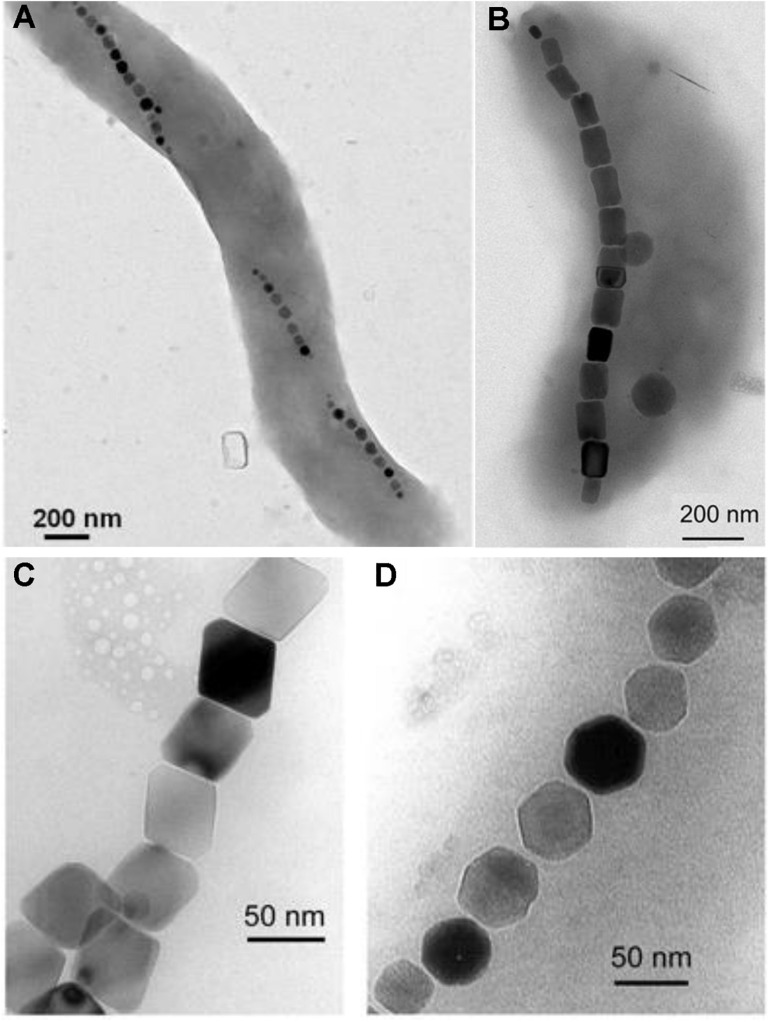
Exemplary transmission electron microscope images of magnetotactic bacteria with magnetosome chains and different magnetosome shapes. **(A)** Example of a magnetotactic *Magnetospirillum* species with chain-like magnetosome arrangement. **(B)** Example of a magnetotactic *Magntovibrio* species with chain-like magnetosome arrangement. **(C)** Magnetite magnetosomes with octahedral and **(D)** cuboctahedral morphologies. Image in part **(A)** was adapted from Alphandéry (2014). Images in part **(B–D)** were adapted from Pósfai et al. (2013).

Magnetotactic bacteria (MTB) form intracellular liposomes, called magnetosomes, filled with crystalline MNPs [mostly magnetite (Blakemore, 1975) or greigite (Mann et al., 1990; Lefèvre et al., 2011)]. MTB also show a chain-like arrangement of the magnetosomes, often positioned mid-cell (Figures 3A,B; Matsunaga et al., 1991; Williams et al., 2012; Bazylinski et al., 2013; Silva et al., 2013), which helps them to passively align their swimming to magnetic field lines (i.e., magnetotaxis; Blakemore, 1975; Bazylinski and Frankel, 2004; Müller et al., 2020). Magnetosomes make MTB susceptible to magnetic fields as low as the earth’s magnetic field (∼0.5 Gauss = 0.05 mT; Blakemore, 1975). It has been also shown that single MTB cells have magnetic moments around 2 × 10^–16^ A m^2^ in magnetic fields below 23 mT (Reufer et al., 2014; Zahn et al., 2017) and can be efficiently sorted and enriched with high-throughput microfluidic methods (Tay et al., 2018). Most magnetosome crystal sizes are between 35 and 120 nm (Bazylinski and Frankel, 2004; Araujo et al., 2015). This results in single-domain states with measureable coercivity rather than superparamagnetism. Furthermore, the linear arrangement and distance of magnetosomes increases the possible range of single-domain particles (Muxworthy and Williams, 2009) and increases cellular motility within external magnetic fields (Pfeiffer and Schüler, 2020).

Most MTB are found in micro-oxic and anoxic environments with an abundance of soluble iron (6–60 μM Fe^2+^; Flies et al., 2005) and show species-specific shape and size properties of MNPs (Lefèvre et al., 2013; Araujo et al., 2015; Uebe and Schüler, 2016; Figures 3C,D). Frequently described species of MTB are *Magnetospirillum* (Blakemore et al., 1979; Matsunaga et al., 1991), *Magnetovibrio* (Silva et al., 2013), *Magnetospira* (Zhu et al., 2010; Williams et al., 2012), *Magnetofaba* (Morillo et al., 2014), and *Magnetococcus* (Lefèvre et al., 2009; Bazylinski et al., 2013). *Magnetospirillum gryphiswaldense* and *Magnetospirillum magneticum* AMB-1 are frequently used to study magnetosome formation and magnetotaxis (Müller et al., 2020).

Magnetotactic bacteria accumulate magnetite up to 2–4% of their dry cell weight (DCW; Blakemore, 1982). It is thought that crystal nucleation proceeds through co-precipitation of soluble Fe^2+^ and Fe^3+^ with subsequent transformation to magnetite (Faivre and Godec, 2015). The thought precursor mineral is ferrihydrite (Faivre and Godec, 2015) that naturally forms nanocrystals below 10 nm (Michel et al., 2007, 2010a), can transform to ordered ferrimagnetic ferrihydrite (Michel et al., 2010a) and is found in the ubiquitous iron-storage protein ferritin (Chasteen and Harrison, 1999; Lewin et al., 2005; Michel et al., 2010b). MTB have a reservoir of special proteins that are located in or close to the magnetosome membrane with up to 120 copies per magnetosome particle (Raschdorf et al., 2018). These proteins are necessary for iron transport (Nies, 2011; Uebe and Schüler, 2016; Uebe et al., 2018; Keren-Khadmy et al., 2020), crystal growth and shape (Arakaki et al., 2010, 2014, 2016; Lopez-Moreno et al., 2017; Nudelman et al., 2018), and intracellular magnetosome arrangement (Scheffel et al., 2006; Abreu et al., 2014; Toro-Nahuelpan et al., 2019). Additionally, some exhibit peroxidase-like activity (Guo et al., 2012; Li et al., 2015), which help to reduce reactive oxygen species from Fenton reactions (Hongping et al., 2015), or prevent accumulation of free radicals (Amor et al., 2020). MTB have magnetosome gene clusters, often with *mam* (magnetosome membrane) and *mms* (magnetic-particle-membrane specific) genes that regulate magnetosome formation (Schübbe et al., 2003; Fukuda et al., 2006). In magnetotactic alphaproteobacteria, magnetosome-related genes are often abbreviated with *mam* or *mms*, in other recently characterized MTB of deltaproteobacteria, nitrospirae and omnitrophica, magnetosome-related genes can be denoted as *mad* or *man* genes (Barber-Zucker and Zarivach, 2017). The magnetosome genes are organized in operons and cluster in a single region, called magnetosome island (Uebe and Schüler, 2016; Barber-Zucker and Zarivach, 2017; McCausland and Komeili, 2020).

In general, magnetosome production requires large amounts of adenosine triphosphate (Yang et al., 2013) and leads to a strong increase of intracellular NADH/NAD^+^ ratio (∼15 after 40 h; Yang et al., 2013). MNP formation is a sensitive process that requires numerous protein-protein interactions and specific environmental conditions. MTB cultivation requires precise media and culture conditions (Ali et al., 2017). A major milestone was the first detailed analysis of magnetosome production in the controlled, microaerobic environment of a bioreactor (Heyen and Schüler, 2003). A maximum magnetite yield of 6.3 mg L^–1^ day^–1^ was found during batch cultivation of *M. gryphiswaldense* with a medium supplemented with 40 μM of iron. However, a cell density of only 0.40 *g* DCW L^–1^ was achieved. Later, researchers were able to produce the current maximum of 9.16 *g* DCW L^–1^ and 178.26 mg magnetite L^–1^ day^–1^ with *M. gryphiswaldense* (Zhang et al., 2011). It is also noteworthy to mention that defined growth media were recently developed that are devoid of uncharacterized and toxic products and yield magnetosome cores with higher iron content (99.8%) compared to common growth media [93.8% (Zhang et al., 2011)] for MTB (Berny et al., 2020).

### Magnetic Non-magnetotactic Cells

#### Erythrocytes

Erythrocytes (red blood cells) are crucial for delivery of O_2_ in the body. They achieve this through their high intracellular hemoglobin content [≤96% of DCW (Weed et al., 1963) or 5.5 mM (Spees et al., 2001)]. Hemoglobin is made of four monomers, each with a covalently bound, Fe^2+^-containing prosthetic heme group (Khoshouei et al., 2017). Three hemoglobin forms are characterized by the state of the iron cation: Deoxyhemoglobin has no bound O_2_ and is paramagnetic due to unpaired electrons of Fe^2+^ (Kurokawa et al., 2018), Oxyhemoglobin has a diamagnetic character due to its bond of Fe^2+^ and O_2_ (Kurokawa et al., 2018), and Methemoglobin contains oxidized Fe^3+^, has paramagnetic behavior and is not able to bind O_2_. Erythrocytes rich in Deoxyhemoglobin or Methemoglobin can be characterized as natural IMCs, because they can be separated from a complex matrix through high magnetic fields (Melville et al., 1975). In fact, erythrocytes are usually separated by intense magnetic field gradients (Toner and Irimia, 2005). It is also worth mentioning that malaria-infected patients show an astonishingly high content in magnetic erythrocytes, which is also used to diagnose the infection with the malaria parasite *Plasmodium falciparum* [e.g., (Zimmerman et al., 2006)]. In its blood stage, *P. falciparum* resides in erythrocytes and digests up to 80% of the present hemoglobin. During the degradation, heme is released into the digestive vacuole, Fe^2+^ is oxidized and after further modifications, cyclic ferriprotoporphyrin IX [Fe(III)PPIX] dimers are produced. The dimers are linked to each other to form a brown, insoluble, crystalline and paramagnetic malaria pigment, called hemozoin (Egan, 2008).

## Proteins and Approaches for the Generation of IMCs

### Iron Importers

Uncontrolled import of iron without a specific intracellular recipient will likely result in disturbances in iron homeostasis (Crichton et al., 2002; Konhauser et al., 2011) and an increase in damaging Fenton reactions (Imlay, 2008; Yamada et al., 2012). However, we want to mention a recent study that showed successful magnetization of algae through overexpression of iron importers (Buck et al., 2015). Although separation of magnetic algae was demonstrated, the researchers highlighted this approach as economically unfeasible, due to low magnetization of algae.

### MTB Proteins

In a hallmark study, researchers generated a recombinant IMC through genomic engineering of the non-magnetic, photosynthetic alphaproteobacterium *Rhodospirillum rubrum* with genes from the magnetotactic alphaproteobacterium *M. gryphiswaldense* (Kolinko et al., 2014). Their final strain harbored 29 relevant magnetosome genes of *M. gryphiswaldense* and was attracted to permanent magnets. The respective intracellular magnetic particles were crystalline magnetite with an average diameter of 24 nm and strongly resembled the magnetosomes of the donor strain. Based on this work, researchers recently aimed to (re)magnetize three non-magnetic *Magnetospirillum* species through a single-step transfer of a vector with > 30 major magnetosome genes from *M. gryphiswaldense* (Dziuba et al., 2020). However, only one strain genomically integrated the vector and produced small, unaligned magnetosomes that hindered efficient magnetotaxis under conditions similar to the geomagnetic field. It is evident that genomic integration of 29 genes and more requires tedious work and single MTB specific vectors have limited applicability for other hosts. In this respect, researchers have identified a possible pool of magnetite interacting MTB proteins. The proteins Mms6 of the *mms6* operon, MamD (Mms7), MamC (Mms13), and MamG (Mms5) of the *mamGFDC* operon strongly bind to magnetite crystals through a charged, hydrophilic C-terminal region, rich in amino acids Glu, Asp, Tyr, Ser, and Thr (Arakaki et al., 2010). Furthermore, this C-terminal region can initiate crystal nucleation and shape of magnetite crystals (Arakaki et al., 2003; Ubago-Rodríguez et al., 2019). The proteins MamG and MamC contain a loop, rich in charged residues that interact with magnetite crystals, which was also found in MmsF and its homologue MamF of the *mamGFDC* operon (Nudelman and Zarivach, 2014; Rawlings et al., 2014; Valverde-Tercedor et al., 2015). Additionally, all contain a hydrophobic glycine-leucine repeat motif, which is common for self-assembling proteins, like silk fibroin (Zhou et al., 2001; Nudelman and Zarivach, 2014).

#### MamD

MamD is a 30.2 kDa magnetosome membrane protein that controls crystal size (Scheffel et al., 2008) and has its Leu-Gly repeat containing N-terminus inside the magnetosome lumen (Arakaki et al., 2003; Nudelman and Zarivach, 2014). However, studies on MamD are scarce and its highly hydrophobic character makes it a hard-to-express protein (Nudelman and Zarivach, 2014).

#### MamG

Similarly to MamD, studies on MamG are scarce and the 8 kDa MamG protein mostly contains hydrophobic transmembrane helices and charged amino acids residues at its ends (Lang and Schüler, 2008; Nudelman and Zarivach, 2014), which makes it also hard to express.

#### MamF/MmsF

The 12.3 kDa MamF protein has a 61% identity with the MmsF protein (Lohße et al., 2011). Both have an integral membrane character with three predicted hydrophobic helices, with the first connecting loop in the magnetosome lumen that contains charged residues for magnetite interaction (Nudelman and Zarivach, 2014). Surprisingly, it was possible to produce both proteins recombinantly in *E. coli* (Rawlings et al., 2014). Even more interesting, the authors found that almost all protein was found in the soluble fraction with an apparent 2-fold mass of 26 kDa under denaturing conditions. They hypothesized extremely stable dimers to be the reason. Cultivation of transfected mesenchymal stem cells that expressed the codon-optimized *mmsF* gene in 35 mM ferric quinate supplemented medium successfully yielded cells with intracellular MNPs (Elfick et al., 2017).

#### MamC

The MamC protein is found in magnetosome membranes and regulates the size of magnetite crystals (Scheffel et al., 2008; Valverde-Tercedor et al., 2015; Peigneux et al., 2016). Due to the hydrophobic character, whole MamC has only been produced as inclusion bodies in *E. coli* (Valverde-Tercedor et al., 2015; Lopez-Moreno et al., 2017). Although the 12.4 kDa protein has two transmembrane helices with a helical connecting loop, rich in charged residues (Nudelman and Zarivach, 2014; Nudelman et al., 2016), it could be a proper candidate for *in vivo* IMC generation. The two acidic residues (Glu66 and Asp70) of the loop match the distance between iron cations in magnetite (Nudelman et al., 2016) and *in vitro* studies on MamC and protein constructs that contained the helical MamC loop resulted in better magnetite size control (Nudelman et al., 2016; Ubago-Rodríguez et al., 2019). This highlighted that only parts of MTB proteins can be used to induce magnetite formation through fusion proteins.

#### Mms6

**FIGURE 4:**
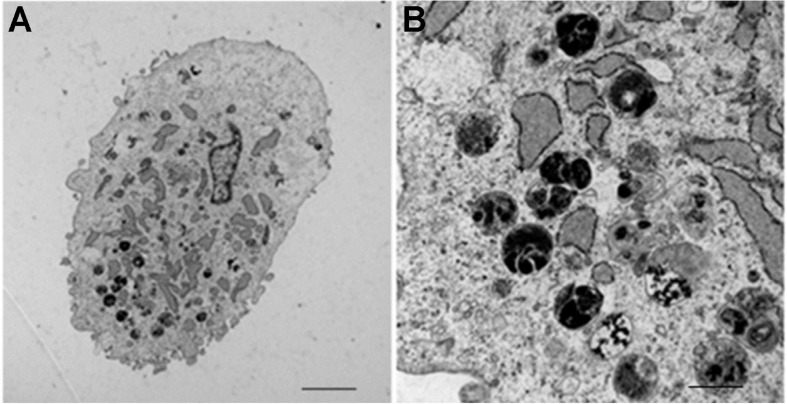
Transmission electron microscope images of *mms6*-transfected mesenchymal stem cells. Pictures were taken after 15 days of cultivation in ferric quinate supplemented medium and show accumulation of membrane-bound, intracytoplasmic electron-dense nanoparticles. **(A)** scale bar = 10 μm. **(B)** scale bar = 100 nm. Images in part **(A,B)** are adapted from Kerans et al. (2018).

Mms6 represents an astonishing and heavily studied magnetosome-associated protein (Staniland and Rawlings, 2016). In *M. magneticum* AMB-1, the native Mms6 protein occurs in two distinct sizes 14.5 kDa and 6 kDa (Nguyen et al., 2016). The smaller 6 kDa version is tightly associated to magnetite crystals in magnetosomes and contains one transmembrane helix between N- and C-terminus (Arakaki et al., 2003; Grünberg et al., 2004; Nudelman and Zarivach, 2014). In addition to magnetite binding, the 14.5 kDa Mms6 version was found to be a binding partner of the important MamA (Nguyen et al., 2016). MamA surrounds magnetosomes and facilitates important protein-protein interactions (Komeili et al., 2004; Yamamoto et al., 2010; Zeytuni et al., 2011). Mms6 is also able to form micelles *in vitro* (200–400 kDa) with a diameter of approximately 10 nm in aqueous solution and the C-terminal, hydrophilic regions exposed (Wang et al., 2012). Astonishingly, addition of iron caused the micelles to form higher order structures (Zhang et al., 2015). Purified Mms6 improved MNP homogeneity during *in vitro* magnetite precipitation (Arakaki et al., 2003; Amemiya et al., 2007; Galloway et al., 2011; Staniland and Rawlings, 2016). Furthermore, experiments with Mms6 and MamC showed that magnetite formation was increased compared to the single protein approaches (Peigneux et al., 2019). Nevertheless, recombinant production of Mms6 in *E. coli* results in inclusion body formation (Amemiya et al., 2007; Prozorov et al., 2007; Bird et al., 2015). However, expression of codon-optimized *mms6* is possible in eukaryotic cells (Zhang et al., 2014; Elfick et al., 2017). Cultivation of *mms6* transfected mouse gliosarcoma 9L cells (Zhang et al., 2014) in 200 μM ferric citrate supplemented medium resulted in formation of intracellular, dark particles as seen by transmission electron microscopy (TEM) images (Zhang et al., 2014). The authors stated an increased iron content of transfected cells together with an increased MRI contrast, but without a decrease in viability. Furthermore, after inoculation of cells into mice, the resulting tumors showed an increased MRI contrast. However, further magnetic studies or iron oxide identification analyses were not performed. A more detailed analysis of mms6 transfected human mesenchymal stem cells also revealed dark aggregates between 10 and 500 nm when cultivated in 34 mM ferric quinate supplemented medium (Elfick et al., 2017; Figures 4A,B). SQUID magnetometry measurements of whole cells highlighted a superparamagnetic character with a saturation magnetization of 23.5 Am^2^ kg^–1^ at 310.15 K (37°C). Based on their measurements, the authors hypothesized the particles to be superparamagnetic magnetite with an average diameter of 12 nm after 2 weeks and approximately 6 × 10^6^ particles per gram biomass after 3 weeks. Thus, Mms6 seems to be a proper candidate for eukaryotic IMC generation. Interestingly, a recent review mentioned unpublished data of this study on the co-transfection of codon-optimized *mms6* and *mmsF* that resulted in less magnetism compared to *mms6* transfection alone (Kerans et al., 2018).

#### MagA

The *magA* gene encodes a putative 46.8 kDa transmembrane iron transporter (Nakamura et al., 1995). Studies with recombinant MagA have focused on increasing MRI contrast of mammalian cells (Zurkiya et al., 2008; Goldhawk et al., 2009; Cho et al., 2014; Rohani et al., 2014; Sengupta et al., 2014). All studies stated that iron supplementation was crucial to achieve MRI contrast and all but one (Cho et al., 2014) reported no adverse effects on cell proliferation and/or cytotoxicity. Interestingly, it was reported that MagA is definitely not involved in magnetosome formation in *M. magneticum* AMB-1 and *M. gryphiswaldense* (Uebe et al., 2012). However, a detailed study with human embryonic kidney (HEK) 293FT-derived clonal cells, that expressed the *magA* gene in 200 μM ferric citrate supplemented medium, described 3–5 nm intracellular particles (Zurkiya et al., 2008). The particles were visualized by TEM and a further extraction and analysis by XRD highlighted similarities to magnetite. An increase in MRI contrast was stated, but faded back to normal values, when induction of the *magA* expression was stopped. The authors hypothesized that the particles were diluted through cell division. However, formation of magnetite through MagA was questioned (Uebe et al., 2012). Magnetite formation could result randomly from high intracellular iron concentrations, due to the ability of MagA to transport iron (Uebe et al., 2012). Recent studies also questioned the general use of MagA to increase MRI contrast. One study reported strong toxic effects of MagA production in murine mesenchymal/stromal cells and kidney-derived stem cells (Pereira et al., 2016). This study also showed that *magA* transfected HEK293TN cells showed comparable accumulation of 5 nm particles, increased intracellular iron concentration and MRI contrast when cultivated in 200 μM ferric citrate supplemented medium. Another study reported an increased intracellular iron content, but no increased MRI contrast of *magA* transfected undifferentiated embryonic mouse teratocarcinoma, multipotent P19 cells when cultured in 250 μM ferric nitrate supplemented medium (Liu et al., 2019). Summarizing, MagA seems to be usable to increase MRI contrast in some mammalian cells, but definite generation of IMCs has not been achieved yet.

### Magnetite Interacting Scaffolds

All of the mentioned MTB proteins (MagA, MamC, MamD, MamF, MmsF, MamG, and Mms6) contain transmembrane regions that limit their usability and overall expression levels. As recently reviewed (Rawlings, 2016), the problems that come with transmembrane regions could be bypassed through *in-silico* methods and protein fusions. This was recently shown for MTB peptides in Adhirons (Rawlings et al., 2015), which represent non-antibody scaffold proteins with high thermal stability (melting temperature ∼101°C) and high soluble expression in *E. coli* (Tiede et al., 2014). The authors used phage display to display Adhirons with two variable binding loops and screened their interaction with cubic magnetite nanoparticles (Rawlings et al., 2015). Through several selection rounds, they identified lysine and histidine residues as key amino acids for magnetite interaction. Furthermore, they showed that only one loop with the sequence QKFVPKSTN was crucial for magnetite interaction and modestly improved magnetite particle appearance in magnetite precipitation reactions. Another study reported the generation of a new non-antibody scaffold protein that resembled an antiparallel, coiled-coil hairpin (Rawlings et al., 2019). The authors further identified magnetite interacting regions of the MTB proteins MamC and MmsF and engineered the scaffold protein to display these regions. Unfortunately, the protein was produced as inclusion body in *E. coli* and needed on-column refolding. In *in vitro* magnetite precipitation experiments, the MmsF construct, but not the MamC construct, produced magnetite particles in size and shape comparable to the native MmsF protein. More studies on such magnetite interacting scaffolds are definitely needed.

### Hemoglobin and Myoglobin

The paramagnetic forms of hemoglobin, namely deoxyhemoglobin, methemoglobin and the crystalline hemozoin, are naturally occurring in erythrocytes, although hemozoin only during malaria infection (Egan, 2008). The paramagnetic behavior can be explained by unpaired electrons of the respective iron cations. When occurring in high amounts, hemoglobins can render the whole cell magnetic (Melville et al., 1975; Egan, 2008). Similarly, myoglobin can become paramagnetic metmyoglobin under oxidizing conditions (Sofla et al., 2013). Myoglobin and each of the four hemoglobin subunits contain a porphyrin ring with an iron at its center (Khoshouei et al., 2017). The rather small size and simple folding pattern of myoglobin and the single hemoglobin subunits make them suitable for recombinant expression. Cytosolic expression of active myoglobin from sperm whale to approximately 10% of total protein was successful in *E. coli* (Springer and Sligar, 1987). Cytosolic production of recombinant hemoglobin above 5% and 7% of total protein content in *E. coli* and *Saccharomyces cerevisiae*, respectively, was also reported (Hoffman et al., 1990; Martínez et al., 2015). However, given the high natural hemoglobin content of erythrocytes, even higher expression levels are needed. Astonishingly, a total intracellular, soluble protein yield of 65% was reported for recombinant overexpression of leghemoglobin (LegH) from soy bean in a proprietary methylotrophic *Komagataella phaffii* MXY0291 strain (Fraser et al., 2018). LegH shows structural similarities to myoglobin (Singh and Varma, 2017). Aside from the *legH* gene, the respective strain also overexpressed all 8 genes of the heme biosynthesis pathway and the transcriptional activator Mxr1 (Fraser et al., 2018) that has been shown to be crucial for the yeast’s methanol pathway and peroxisomal enzymes (Lin-Cereghino et al., 2006). Unfortunately, data on the strain’s physiology, cultivation and magnetic behavior is not available. However, this proofs that active folding and high titers of heme-containing globins are not limited to mammalian cells as long as the necessary genetic prerequisites are given. Nevertheless, high intracellular heme-protein content for cell magnetization might be rather limited to erythrocytes. Furthermore, based on magnetic properties, heme proteins will never result in cell magnetization comparable to *in vivo* magnetite production. However, it must be mentioned that extracellular triggers can be used to increase magnetic susceptibility of heme-protein-rich cells, as shown for myoglobin-rich cardiomyocytes (Sofla et al., 2013). Naturally, the cytoplasmic protein fraction of these cells consists only of 5–10% of the heme-protein myoglobin (Berridge et al., 2013). The use of the highly oxidizing agent NaNO_2_ transformed all myoglobin to paramagnetic metmyoglobin to generate magnetic cardiomyocytes (Sofla et al., 2013).

### Ferritin

Ferritins are a family of highly conserved protein nanostructures that hold and sequester iron atoms. Theoretically, ferritins store up to 4,500 iron atoms as iron oxides in their inner cavity, but typically an average of 2,000 iron atoms are present (Jutz et al., 2015). All ferritins have an α-helix structure with strong helix-helix interactions that make the proteins very stable (Theil, 1987; Martsev et al., 1998). Ferritins usually consist of 24 subunits and have a size around 450 kDa in the apo-ferritin state (Theil, 1987). Ferritin cavities can have diameters between 5 and 8 nm (Theil, 2012). Mammalian and plant ferritins consist of a certain ratio of H- and L-subunits and are soluble in the cytoplasm and plastids. The H-subunit has an iron center, where fast conversion of Fe^2+^ to Fe^3+^ by O_2_ or hydrogen peroxide occurs (Lawson et al., 1989). The L-subunit contributes to the nucleation of the iron core and protein stability (Santambrogio et al., 1992). Bacteria have two types of ferritins, the archetypal ferritins with 24 identical H-type subunits and the heme containing bacterioferritins, also consisting of 24 H-subunits. Bacterioferritins (∼450 kDa) and bacterial non-heme ferritins (∼470 kDa) from *E. coli* are essential for growth and store around 2,500–2,700 iron atoms (Andrews et al., 2003). The iron oxide core structure and crystallinity can vary from amorphous to nanocrystalline and often contains a material similar to ferrihydrite (Lewin et al., 2005; Michel et al., 2010b) that naturally forms nanocrystals below 10 nm (Michel et al., 2007).

Due to their iron oxide storage capacity, ferritins have been studied as intrinsic MRI contrast agents (Naumova and Vande Velde, 2018). Researchers have been also eager to use ferritin for *in vitro* production of MNPs. Early *in vitro* studies mainly produced maghemite MNPs with horse spleen ferritin (Meldrum et al., 1992; Wong et al., 1998) and human H-chain ferritin (hHf; Uchida et al., 2006). Later, studies with hHf succeeded in magnetite MNP production (Cao et al., 2010; Walls et al., 2013). The authors found a surprisingly high number of iron atoms (8,400) in the hHf cavity (Walls et al., 2013). Convincingly, they argued that magnetite is denser than other iron oxides and that the 7.5 nm wide cavity of hHf would fit nearly 9,000 Fe atoms (Walls et al., 2013). Based on these successful *in vitro* results, *in vivo* formation of magnetic ferritins to increase intrinsic cellular MRI contrast and to generate IMCs seems possible. This was tried with mammalian HEK293T cells (Kim et al., 2012). First, the authors found that cultivation in 3 mM ferrous ammonium sulfate supplemented medium and recombinant expression of hHf resulted only in minor intracellular iron accumulation and no cell magnetization. However, additional co-expression of the divalent metal transporter 1 (DMT1) increased intracellular iron content significantly. Only then, the cells were attracted to permanent magnets by 30 μm s^–1^. The engineered cells were also retained to 25% on a MACS column, but unfortunately no further analyses were performed to characterize the source of cellular magnetism. Another study reported an almost 3-fold increase in magnetic binding of *S. cerevisiae* cells through expression of a mutated form of the thermostable, homo-multimeric ferritin of *Pyrococcus furiosus* (Matsumoto et al., 2015). The authors assumed that higher ferritin iron content would increase ferritin magnetization, as recently shown *in vitro* for hHf (Walls et al., 2013) and therefore selected ferritin mutants with increased iron binding. Their most promising mutant ferritin candidate, L55P, induced an almost 2-fold greater iron accumulation than the wild-type (Matsumoto et al., 2015). Although their mutant ferritin showed paramagnetic behavior at 5 K in SQUID magnetometry measurements and around 60% of mutant-expressing yeast cells were retained on a magnetic column, MRI analysis of the respective yeast cells showed no significant difference of the signals when normalized to the intracellular iron content. This implied no formation of a distinct magnetic iron oxide form, but rather an increase of magnetization through intracellular iron. Similar results were found for an *E. coli* knockout strain without the native ferritin-clan genes (*ftnA*, *bfr*, and *dps*) and iron exporters (*rcnA, fieF, and zntA*) that overexpressed a ferritin (FtnA) double-mutant (H34L, T64I) together with iron importers (Liu et al., 2016). SQUID magnetometry measurements of the mutant cells indicated a paramagnetic character and around 70% of cells were retained on a MACS column. However, no distinct formation of magnetic iron oxides in the ferritin cores was stated.

Together, these results highlight that overexpression of ferritins and mutant ferritins with higher iron binding capacity is a promising starting point, but more sophisticated methods to induce *in vivo* magnetite formation with ferritins are needed. One possible approach was recently shown with a ferritin-M6A chimera (Radoul et al., 2016). The authors fused the charged C-terminal region (M6A) of the MTB Mms6 protein that strongly binds magnetite and is crucial for magnetite biomineralization (Arakaki et al., 2003; Ubago-Rodríguez et al., 2019) to the mouse H-ferritin subunit (Radoul et al., 2016). Ferritin-M6A formed ∼440 kDa structures that were found to be ∼12 nm protein particles with 24 (∼25.8 kDa) monomers able to bind iron. Expression of ferritin-M6A in rat glioma C6 cells resulted in increased iron content, and *in vivo* analysis of tumor xenografts in mice that expressed ferritin-M6A showed also increased MRI contrast. Summarizing, engineered ferritins have the potential to magnetize cells, their easy expression in various hosts increases their applicability, but definite magnetite formation for strong magnetization of cells requires further research.

### Encapsulins

Prokaryotic, proteinaceous proteins, called encapsulins, were structurally described to form large icosahedral shells by intracellular self-assembly of monomers that function as a minimal compartment (Sutter et al., 2008). These nanocompartments are suggested to play an important role in many microbial organisms and form capsids around 25–35 nm in diameter (Nichols et al., 2017). Similar to ferritin, they are pH resistant and temperature stable. However, they have a native set of cargo molecules that are packaged into the shells via a specific short terminal peptide tag that can be used to target other proteins to the shell (Cassidy-Amstutz et al., 2016). Encapsulins were not only successfully produced in prokaryotes (McHugh et al., 2014; Lagoutte et al., 2018), but also in eukaryotes (Lau et al., 2018; Sigmund et al., 2018), which makes them versatile tools for localized reactions. An interesting candidate for the generation of IMCs could be the encapsulin of the gram-negative bacterium *Myxococcus xanthus*. The *M. xanthus* encapsulin consists of a shell protein (EncA; 32.5 kDa) and contains three internal proteins (EncB; 17 kDa; EncC; 13 kDa; EncD; 11 kDa; McHugh et al., 2014). Built from 180 EncA monomers, the final encapsulin has an average diameter of 32 nm with an internal diameter of 26 nm. Furthermore, the authors showed that EncB and EncC contain ferritin-like domains attached to the inner encapsulin surface. They further found ∼5 nm electron-dense granules inside the encapsulin and calculated that it could potentially hold up to 30,000 iron atoms, which marks an approximate 10-fold increase compared to ferritin. However, the authors gave no indication on the iron phase inside the encapsulin (McHugh et al., 2014). In a recent study, the authors expressed the EncA protein of *M. xanthus* together with EncB or EncC in mammalian HEK293T cells (Sigmund et al., 2018). They reported efficient iron encapsulation for MRI contrast and MACS. Cell viability was not negatively affected. Unfortunately, the authors performed no further magnetometry investigations on the cells. Interestingly, they tried to use the beneficial magnetite interacting ability of Mms6 and MamD (Mms7) through fusion of the C-terminal regions to EncA, B, or C. However, they reported no beneficial impact on iron loading compared to the native encapsulin proteins. Nevertheless, they demonstrated that encapsulins are potential candidates for recombinant IMC generation.

### Iron-Sulfur Cluster Proteins

Iron-sulfur cluster proteins have a crucial role in various physiological processes. In the oxidized state, [2Fe-2S] cluster proteins have Fe^3+^ atoms, leading to 5 unpaired electrons at each iron. It has been shown that the two spin-bearing iron centers are coupled to each other through an exchange-driven antiferromagnetic coupling mechanism (Ali M.E. et al., 2015). Some iron-sulfur cluster proteins can be expressed to high titers of 30% total soluble protein (Ta and Vickery, 1992; Gubernator et al., 2003). However to our knowledge, magnetic separation techniques have not been performed with iron-sulfur cluster proteins until recently. Migratory animals are thought to have special, magnetically sensitive receptors that help in navigation on their journeys. Recently, a potential protein candidate was found (Qin et al., 2016). The authors demonstrated that the protein MagR (magnetic receptor), a homologue of the Iron-Sulfur Cluster Assembly 1 (Isca1) protein, when in complex with another protein, cryptochrome (Cry), formed a multimeric protein complex that responded to magnetic fields *in vitro*. Based on a structural analysis of MagR multimers and the MagR/Cry complex, the authors argued that the magnetic behavior could result from alignment of MagR monomers (Qin et al., 2016). Additionally, they were able to enrich the MagR protein and MagR/Cry complex, respectively, from a complex matrix with a magnet and non-magnetized iron beads (Qin et al., 2016). Later, this method was also shown to be effective to capture MagR fusion proteins from a complex matrix (Jiang et al., 2017; Wang L. et al., 2019). However, the physical capabilities of this protein complex were recently questioned (Meister, 2016; Winklhofer and Mouritsen, 2016) and the putative magnetic properties have been still under review. When MagR-membrane channel constructs were subjected to magnetic stimuli in HEK cells, they were not able to induce significant membrane channel activity in a magnetic field (Pang et al., 2017; Wang G. et al., 2019). Therefore, usability of MagR for IMC generation might be farfetched at this point and research on MagR and similar iron-sulfur cluster proteins is needed.

## Envisioned Applications of IMCs

### Recombinant Production of Magnetosomes and MNPs

Magnetic nanoparticles have a wide application range in medicine (Li et al., 2016), but their production has to be cost-efficient. Although research on the mass production of MTB has brought fruitful results, common industrially relevant cellular organisms, like *E. coli* or *S. cerevisiae*, are easier to cultivate to high cell densities. Therefore, transformation of non-magnetic cells to magnetosome-producing cells is a quest (Kolinko et al., 2014; Uebe and Schüler, 2016). Recently, a patent has been granted (US9913918B2) that protects the heterologous expression of gene cassettes that comprise several MTB operons (Kolinko et al., 2014). Another set of patents describing the introduction of MTB into eukaryotic cells as artificial endosymbionts was recently granted (e.g., US8828681B2). Briefly, it is stated that MTB are genetically modified to survive conditions in the eukaryotic cytosol that further provides sufficient nutrients and micro-oxic conditions for magnetosome formation. Technically, the resulting eukaryotic cell has to be regarded as an IMC. Although not stated specifically, successful generation of MTB-endosymbiotic yeasts could be one way to facilitate mass production. Given the production of superparamagnetic MNPs with an estimated size of 12 nm in *mms6* transfected human mesenchymal stem cells (Elfick et al., 2017), production of MNPs, as side products in costly mammalian cell cultures, could be also envisioned, especially in pharmaceutically-relevant mammalian antibody production cell lines.

### MRI Contrast

In recent years concerns have arisen surrounding the long-term safety of current Gadolinium(III)-based MRI contrast compounds (Runge, 2017), and this has spurred research into alternatives. It was shown that MTB can produce positive MRI contrast (Benoit et al., 2009). Additionally, although application in humans will be a future challenge, a number of studies focused on the improvement of cellular MRI contrast through genetic engineering of cells through MTB proteins (Zurkiya et al., 2008; Goldhawk et al., 2009; Cho et al., 2014; Rohani et al., 2014; Sengupta et al., 2014; Elfick et al., 2017) and ferritins (Cohen et al., 2005; Matsumoto et al., 2015; Liu et al., 2016; Radoul et al., 2016).

### Magnetic Drug and Cell Delivery

Targeted drug delivery refers to predominant drug accumulation at a target zone (Torchilin, 2000). A small number of publications dealt with the use of MTB for magnetic drug and cell delivery in cancer treatment (Martel et al., 2009; Felfoul et al., 2016). In tissue engineering, intrinsically magnetic mesenchymal stem cells could be used to repair tissue damage by tracking, targeting and local long-term retention of cells at the specific site of damage (Kerans et al., 2018).

### Magnetic Hyperthermia in Cancer Treatment

Magnetic hyperthermia is based on the concept that MNPs generate heat when exposed to an external, alternating magnetic field (Carrey et al., 2011; Beik et al., 2016). In hyperthermia therapy, heat is increased within or near a tumor to induce cellular changes including protein denaturation, damage to the cytoskeleton and disruption of DNA repair that lead to cell death (Jha et al., 2016). Studies with *M. gryphiswaldense* and *M. magneticum* AMB-1 showed that IMCs present proper tools for magnetic hyperthermia (Alphandéry et al., 2011; Gandia et al., 2019). Additionally, a recent review mentioned the potential use of intrinsically magnetic mesenchymal stem cells for localized magnetic hyperthermia treatment, but also highlighted that future studies have to generate a sufficient amount of intracellular MNPs (Kerans et al., 2018).

### IMCs in Bioprocess Engineering

Intrinsically magnetic cells are interesting for immobilized, whole cell catalysis that often uses magnetic beads for cell immobilization (Al-Qodah et al., 2018). With IMCs, immobilization of cells can be solely done through magnetic fields, which eliminates chemical coupling to bead surfaces.

Some cells, especially mammalian cells, are very sensitive to shear forces, which could be minimized through magnetic harvesting. Once mammalian cells exhibit magnetic behavior, they can be simply harvested through a magnetic field that attracts cells to the walls or bottom of the vessel, rather than using stressful centrifugation and filtration. The same approach might be applied for cell retention in continuous bioprocessing.

## Outlook

Simple overexpression of iron importers to increase intracellular iron concentrations is not the method of choice to magnetize cells. Free iron cations induce Fenton reactions and disturb iron homeostasis and show only little magnetic properties (Buck et al., 2015). Also the use of heme-proteins to magnetize cells seems to be limited. Researchers showed that heme-proteins can be expressed to high intracellular titers, but their magnetic character is only sufficiently present under oxygen-free or highly oxidizing conditions (Sofla et al., 2013). Moreover, the example of erythrocytes shows that extensive amounts of heme-proteins must be present for cell magnetization, which clearly limits recombinant approaches. Similarly, MagR might not be usable for IMC generation, since current *in vivo* studies failed. Better prospects for IMC generation are clearly envisioned for MTB proteins, ferritins and encapsulins.

Magnetotactic bacteria accumulate magnetic minerals to around 3% of their DCW, their magnetosomes show single-domain magnetic behavior, have sizes between 35 and 120 nm and are chain-like arranged. MTB present the golden standard of an IMC. Genetic engineering of non-magnetic hosts like *R. rubrum* with 29 magnetosome genes from *M. gryphiswaldense* yielded a magnetic cell (Kolinko et al., 2014). This study also yielded the largest recombinantly produced MNPs (∼24 nm) in an initially non-magnetic cell. However, transferring a large pool of MTB proteins to non-magnetic hosts does not necessarily yield IMCs, as shown for non-magnetic *Magnetospirillum* species (Dziuba et al., 2020). We also argue that expression of MTB proteins might be tricky in common industrial hosts, like the bacterium *E. coli*. The work of Juodeikis in 2016 presents a good overview on soluble and insoluble production of various MTB proteins in *E. coli* (Juodeikis, 2016). Although recombinant overexpression of MTB proteins (e.g., MagA, MmsF, or Mms6) showed better results in eukaryotic hosts, probably due to the better equipped protein folding machinery and posttranslational protein modifications (Barber and Rinehart, 2018), we argue that increasing the soluble protein expression must be a priority to successfully generate IMCs with MTB proteins. The amino acid sequence QKFVPKSTN, crucial for magnetite interaction and improvement of magnetite particle appearance, seems to be especially promising for these approaches (Rawlings et al., 2015). Furthermore, we hypothesize that the Mms6 protein marks a proper starting point for IMC generation (Elfick et al., 2017).

Compared to MTB proteins, ferritins are limited in their possible MNP size (∼5–8 nm). Therefore, ferritins could be the proteins of choice for *in vivo* production of superparamagnetic MNPs. Their ubiquitous occurrence and soluble character makes them perfect for recombinant expression. Native ferritins naturally contain no magnetite, but rather amorphous or crystalline ferrihydrite that forms crystals below 10 nm. However, it was already proven that ferritins can form relatively pure magnetite *in vitro* (Walls et al., 2013). Weak cell magnetization with ferritins was already achieved in bacteria (Liu et al., 2016), yeasts (Matsumoto et al., 2015), and mammalian cells (Kim et al., 2012), but unfortunately no study reported magnetite *in vivo*. Radoul et al. (2016) presented a promising ferritin-M6A fusion to increase *in vivo* magnetite formation. It would be interesting to investigate how this ferritin-M6A fusion can be improved in iron loading through mutagenesis.

Similar to ferritins, encapsulins provide a protecting shell for iron oxide formation, but research on encapsulins has only started. Through their ability to form >20 nm nanocompartments, good expression levels in prokaryotes and eukaryotes and protein targeting inside these nanocompartments, their usability for localized magnetite forming reactions seems promising. Native *M. xanthus* encapsulin weakly magnetized mammalian cells *in vivo* only through the amount of iron stored in its inner core (Sigmund et al., 2018). The authors also tried to improve the encapsulin’s iron storage abilities through fusions of MTB protein peptides, but this resulted in no improvement. However, we argue that further studies should be performed with encapsulins. It might be interesting to investigate how hypoxic conditions affect iron oxide content of encapsulins and if engineered encapsulin proteins could be used to produce rather big, single-domain iron oxide particles.

## Author Contributions

AP read the literature and drafted the manuscript. OS supervised and corrected the manuscript draft. All authors contributed to the article and approved the submitted version.

## Conflict of Interest

The authors declare that the research was conducted in the absence of any commercial or financial relationships that could be construed as a potential conflict of interest.

## Abbreviations

DCW: dry cell weight
hHf: human H-chain ferritin
IMCs: intrinsically magnetic cells
M6A: charged C-terminal region of the MTB Mms6 protein
MACS: magnet-assisted cell separation
mam: magnetosome membrane
mms: magnetic-particle-membrane specific
MNPs: magnetic nanoparticles
MPI: magnetic particle imaging
MRI: magnetic resonance imaging
MTB: magnetotactic bacteria
P + MNP: protein coupled to magnetic nanoparticles
SQUID: superconducting quantum interference device
T_*C*_: Curie-temperature
TEM: transmission electron microscopy
T_*N*_: Néel-temperature
VSM: vibrating sample mode
XRD: X-ray diffraction.
